# Measurements of heterotypic associations between cluster of differentiation CD74 and CD44 in human breast cancer-derived cells

**DOI:** 10.18632/oncotarget.20922

**Published:** 2017-09-14

**Authors:** Hussain Al Ssadh, Patrick S. Spencer, Waleed Alabdulmenaim, Rana Alghamdi, Inamul Hasan Madar, Jose M. Miranda-Sayago, Nelson Fernández

**Affiliations:** ^1^ School of Biological Sciences, University of Essex, Wivenhoe Park, Colchester, Essex CO4 3SQ, United Kingdom; ^2^ Pathology Department, College of Medicine, Qassim University, Qassim, Saudi Arabia; ^3^ King Abdulaziz University, Rabigh Campus, Rabigh, Saudi Arabia; ^4^ Department of Biotechnology and Genetic Engineering, Bharathidasan University, Tiruchirappalli, 620024, Tamil Nadu, India

**Keywords:** CD74, CD44s, CD44v, co-localization

## Abstract

Interactions between pairs of membrane-bound receptors can enhance tumour development with implications for targeted therapies for cancer. Here we demonstrate clear heterotypic interaction between CD74 and CD44, which might act in synergy and hence contribute to breast cancer progression. CD74, a type II transmembrane glycoprotein, is a chaperone for MHC class II biosynthesis and a receptor for the MIF. CD44 is the receptor for hyaluronic acid and is a Type I transmembrane protein. Interactions between CD74, MIF and the intra-cytoplasmic domain of CD44 result in activation of ERK1/2 pathway, leading to increased cell proliferation and decreased apoptosis. The level of CD44 in the breast tumor cell lines CAMA-1, MDA-MB-231, MDA-MB-435 and the immortalized normal luminal cell line 226LDM was higher than that of CD74. It was also observed that CD74 and CD44 exhibit significant variation in expression levels across the cells. CD74 and CD44 were observed to accumulate in cytoplasmic compartments, suggesting they associate with each other to facilitate tumour growth and metastasis. Use of a novel and validated colocalisation and image processing approach, coupled with co-immunoprecipitation, confirmed that CD74 and CD44 physically interact, suggesting a possible role in breast tumour growth. This is the first time that CD74 and CD44 colocalization has been quantified in breast cancer cells using a non-invasive and validated bioimaging procedure. Measuring the co-expression levels of CD74 and CD44 could potentially be used as a ‘biomarker signature’ to monitor different stages of breast cancer.

## INTRODUCTION

Simultaneous expression of the cluster of differentiation (CD) 74 and CD44 has been identified in several types of cancer and is believed to play a role in tumour development [1–3]. CD74, is a nonpolymorphic type II integral membrane glycoprotein expressed by antigen-presenting cells [4]. CD44, a type I transmembrane glycoprotein, and member of the cartilage link protein family is expressed in most cell types and is a receptor for hyaluron and osteopontin [5]. CD44 is involved in cell-to-cell and cell-extracellular matrix interactions, cell adhesion and migration. In a previous study, we showed that adhesion of tumour cells on the reconstituted membrane matrix Matrigel increases when CD74 and CD44 are overexpressed [6]. This was accompanied by increased expression of membrane-bound and soluble proteins involved in cell adhesion and cancer metastasis. In breast cancer cells, the lipid-modified glycoprotein, WNT5A, inhibits metastasis and alters the splicing of CD44 [7]. Recently, it has been shown that CD74 and CD44 promote actin polymerization via RHOA-mediated CFL1 phosphorylation; it is thought that this interaction contributes to tumour metastasis [8].

CD74 protein exists in a number of isoforms which are post-translationally glycosylated [1]. The best known function of CD74 is as a chaperone involved in stabilizing nascent human leucocyte antigen (HLA)-DR class II αβ-heterodimers [9–11]. By means of two motifs in its cytosolic N′ -terminus, Ii directs class II nonamers toward endosomal compartments in which peptide loading occurs. Lastly, aminoacid (aa) 81-104 of Ii, termed CLIP (class II-associated invariant chain peptide), which is bound in the class II peptide binding groove, prevents loading of endoplasmic reticulum (ER)-resident peptides onto HLA class II. The expression of MHC class II in tumors, including colorectal and breast carcinomas, has been previously observed [12]. HLA-DR proteins in breast epithelium, which normally lacks MHC II expression, may be induced by cytokines or hormones [13]. Coordinate expression of HLA-DR, CD74 and HLA-DM by tumour cells is thought to be an indicator of improved prognosis in breast carcinoma. Several groups have suggested that the expression of HLA molecules improves immunogenicity of tumour cells and induce an anti-tumour T-cell response [14–16].

CD74 is also the ligand for the innate cytokine macrophage migration inhibitory factor (MIF), which binds with high affinity to the extracellular domain of cell surface CD74 [17, 18]. It also interacts with amyloid precursor protein and suppresses amyloid-β protein synthesis [19]. In immune cells, MIF binding to CD74 induces a signaling cascade regulating cell proliferation and survival [20]. CD74 lacks intracellular signalling domains, with MIF-induced extracellular signal-regulated kinase (ERK) signalling reliant upon CD44 [21, 22]. To enable interaction with CD44, CD74 is modified by the addition of chondroitin sulfate, permitting formation of a molecular complex between MIF, CD74 and CD44 [23, 24].

CD44 has been the subject of research interest because of its potential role in breast cancer. CD44 activates and inhibits oncogenic signalling in response to extracellular signals [25, 26]. Expression of CD44 is upregulated in premalignant lesions and is associated with particular cancer types, including prostate cancer, head and neck squamous-cell carcinoma, central nervous system malignancies, respiratory track malignancies, melanoma and breast cancer [27–30]. The CD44 gene undergoes extensive alternative splicing of multiple variable exons that are positioned in a cassette in the middle of the gene [31]. Present in most cell types, CD44s, the standard splice isoform, comprises exons 1-5 and 16-20; CD44v isoforms are splice variants containing variable exons. It is possible that expression of alternative CD44 exons is associated with tumour progression. However, expression of CD44 in human breast tumors is correlated with both favourable and unfavourable clinical outcomes [25, 32].

Previously, we have demonstrated that high expression of signal transducer and activator of transcription 1 (STAT1) and CD74 is associated with triple-negative breast cancer [6]. The present study sought to characterize the cell-surface and total protein expression of CD74 and CD44 in three human breast cancer cell lines types (CAMA-1, MDA-MB-231, MDA-MB-435) and in normal luminal 226LDM cells. Heterotypic interaction between CD74 and CD44 is for the first time demonstrated at a quantitative level using a novel colocalization and image processing approach coupled with co-immunoprecipitation (Co-IP) techniques.

## RESULTS

### Identification and quantification of CD74 and CD44

**Figure 1:**
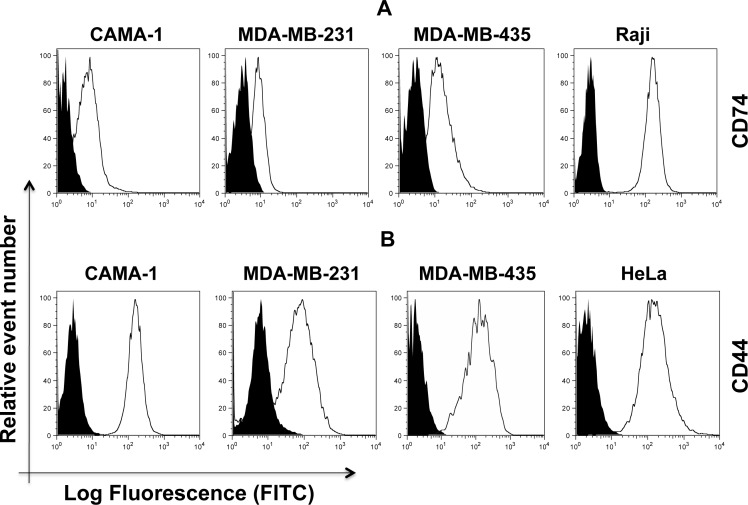
Cell surface expression of CD74 (upper panel, **(A)** and CD44 (lower panel, **(B)** on CAMA-1, MDA-MB-231, MDA-MB-435, Raji and HeLa cells. All cells were cultured in the appropriate media and were acquired by flow cytometry using By2 (anti-CD74) and 156-3C11 (anti-CD44). Empty histograms represent the aforementioned cell lines labeled with anti-CD74 and anti-CD44 antibody. Black-filled histograms show the isotypes serving as negative controls. Cells were labelled using FITC-labelled secondary anti-mouse antibody. Expression levels were analyzed by flow cytometry (Aria cell sorter) and FlowJo 8.8.6 software was used to analyze the data. Mean fluorescence intensity (MFI) values were measured based on geometric means. Mouse IgG was used as a negative control. Data are representative of three independent experiments.

The cell surface expression of CD74 and CD44 was appraised in CAMA-1, MDA-MB-231, MDA-MB-435, Raji and cervical cancer HeLa cells. Monocytes, Raji cells and HeLa cells were used as a positive control, since CD74 in monocytes and CD44 in Raji cells and HeLa cells are expressed in high amounts. Non-permeabilized cells were stained with By2 (anti-CD74) and 156-3C11 (anti-CD44) antibodies, followed by addition of RAM-FITC, as a secondary antibody. CD74 and CD44 were clearly detected in the plasma membrane of CAMA-1, MDA-MB-231 and MDA-MB-435 cells (Figure 1).

**Figure 2:**
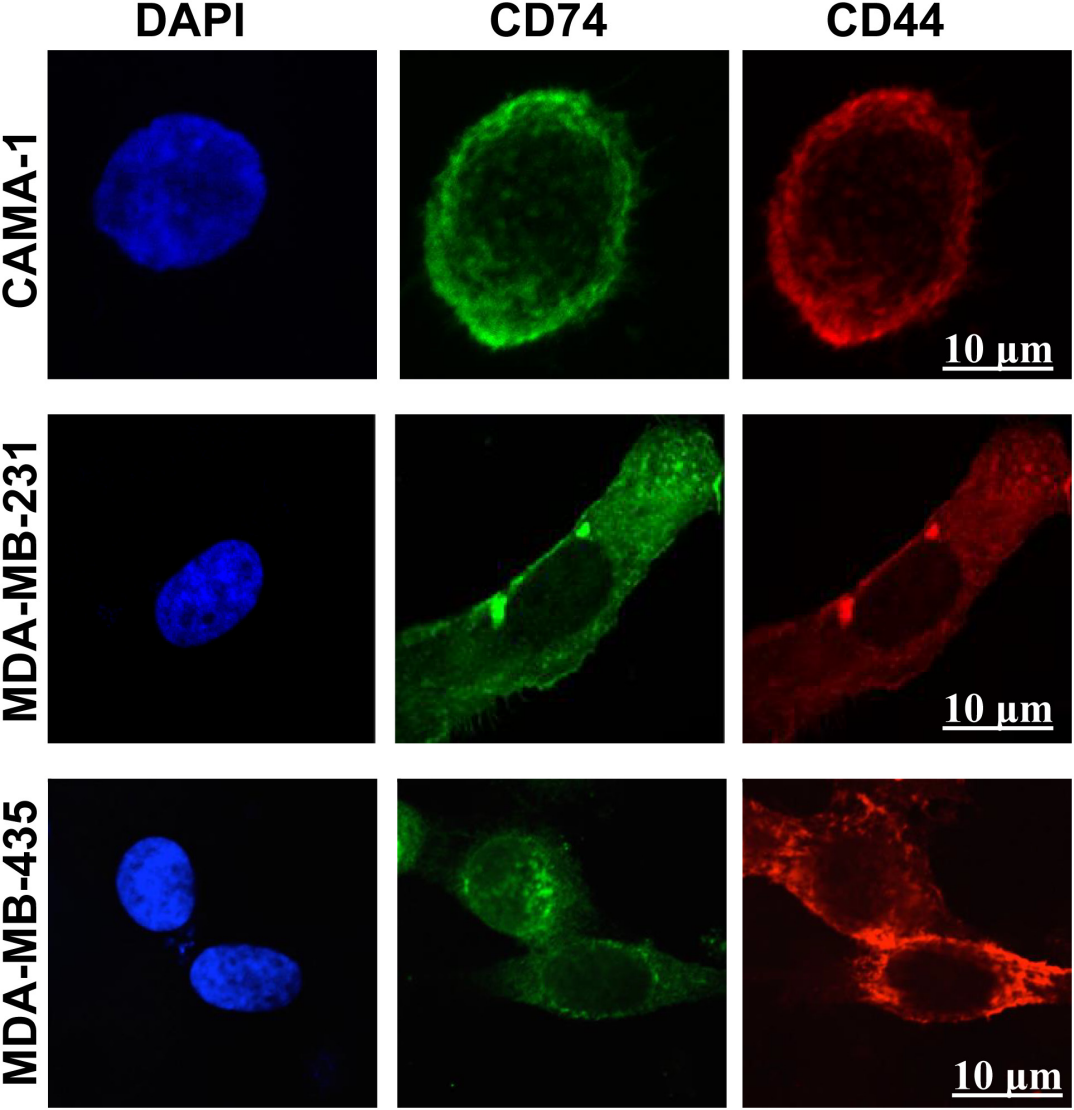
Confocal laser scanning microscopy images of intracellular staining of CD74 and CD44 in CAMA-1, MDA-MB-231 and MDA-MB-435 cells The cells were cultured in LabTek 8-well chambers at a density of 6 × 10^3^ cells per well overnight. CD74 was labelled with Alexa Fluor 488 (green) and CD44 with Alexa Fluor 555 (red). 4′, 6-diamidino-2-phenylindole was used for nuclear staining (blue). Fluorochromes were acquired separately to evaluate the expression of CD74 and CD44 using the image-analysis software platform Fiji. Photomicrographs are representative of three independent experiments. Scale bar 10 μm.

Laser scanning confocal microscopy, using different wavelengths, was used to visualize intracellular expression of CD74 and CD44 molecules. The CAMA-1, MDA-MB-231 and MDA-MB-435 cell lines all expressed CD74 and CD44 in intracellular compartments (Figure 2).

### Immunoblot analysis of CD74 and CD44 proteins

**Figure 3:**
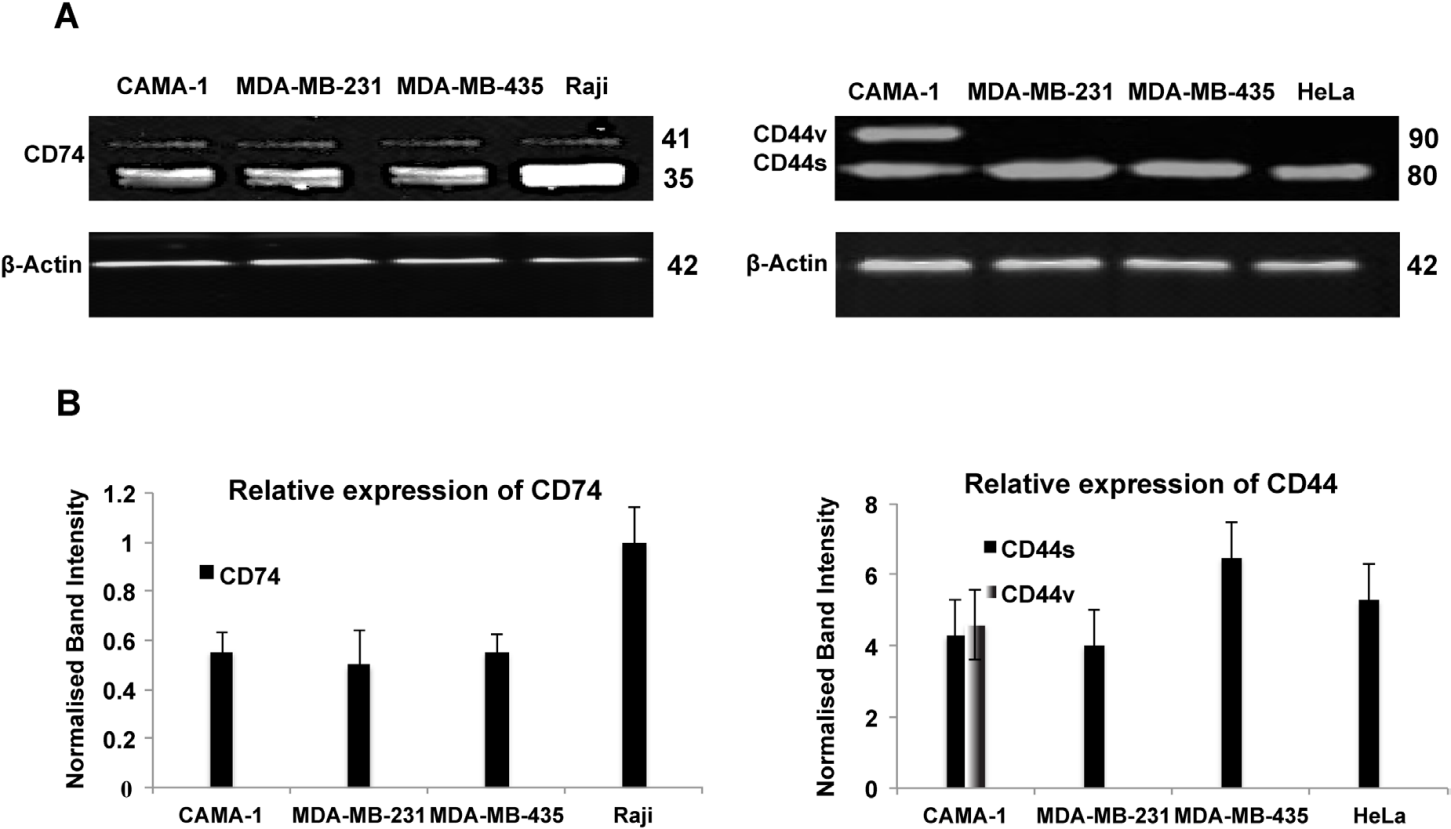
Western blot analysis of CD74, CD44 and β-actin expression in the CAMA-1, MDA-MB-231, MDA-MB-435 and Raji cells **(A)** Primary monoclonal antibodies Poly6221 (anti-β-actin as loading control), By2 (anti-CD74) and 156-3C11 (anti-CD44) were used. β-actin was detected at a molecular weight of 42 kDa; CD74 isoforms were detected at molecular weights 35 and 41 kDa, whereas CD44 isoforms were detected at molecular weights 80 and 90 kDa. **(B)** CD74 and CD44 levels are normalized against β-actin. To account for the difference in protein loading during the experiment, the percentage of expression was calculated after the intensity of each band was adjusted according to its respective β-actin band intensity using the Image Studio Lite software (LI-COR Biosciences). Figures depict representative samples from triplicate experiments displayed as composites of multiple Western blots.

In order to ascertain that the receptors detected by the antibodies corresponded to CD74 and CD44, protein expression in CAMA-1, MDA-MB-231, MDA-MB-435, Raji and HeLa cells was studied by Western blot analysis using By2 (anti-CD74), 156-3C11 (anti-CD44) and Poly6221 (anti β-Actin). β-actin was used as a loading control. CD74, CD44 and β-actin have molecular weights of 33-41 kDa, 80-90 kDa and 42 kDa, respectively (Figure 3). All cell lines expressed two CD74 isoforms, the p35 and p41 isoforms (Figure 3A; right panel). CAMA-1 cells expressed two isoforms of CD44, with molecular weight of 80 kDa and 90 kDa (Figure 3A; left panel). Figure 3B shows the expression percentages of CD74 and CD44, with expression data normalized to the β-Actin reference.

### Expression of CD74 and CD44 in normal breast cells

**Figure 4:**
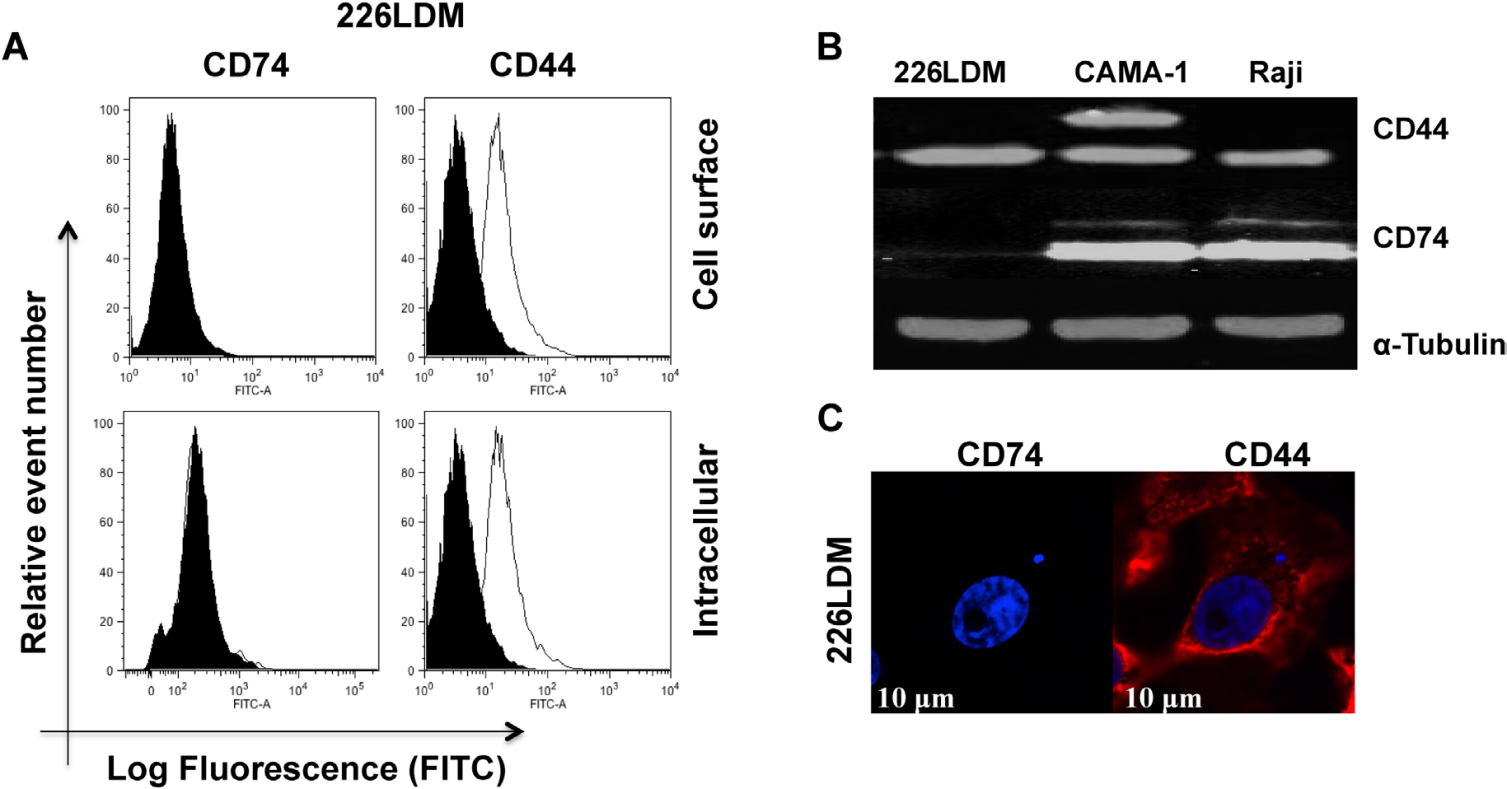
The expression of CD74 and CD44 receptors in immortalized normal breast luminal cells (226LDM) **(A)** Cell-surface and intracellular expression of CD74 and CD44 was acquired by flow cytometry using By2 (anti-CD74) and 156-3C11 (anti-CD44). Black-filled histograms represent the 226LDM cells stained with indicated antibody. Empty histograms show the isotype as negative controls. **(B)** Total protein of CD74 and CD44 was detected by Western blotting, and α-tubulin was used as a loading control. **(C)** Confocal images of CD74 and CD44 in 226LDM cells stained intracellularly: CD74 was labelled with Alexa Fluor 488 (green) and CD44 with Alexa Fluor 555 (red). Figures depict representative samples from duplicate experiments. Scale bar 10 μm.

We examined the expression of CD74 and CD44 in immortalized normal luminal 226LDM cells. Cell-surface and intracellular expression of both proteins was assessed by flow cytometry (Figure 4A). Total expression level of CD74 and CD44 was detected by Western blot analysis with α-tubulin used as a loading control (Figure 4B). Confocal laser-scanning microscopy was utilized to study the intracellular staining of CD74 and CD44 in 226LDM cells (Figure 4C). The results show that 226LDM cells do not express detectable levels of CD74. However, the cells express CD44 in intracellular compartments, with weak cell surface expression.

### Colocalization analysis of CD74 and CD44

**Figure 5:**
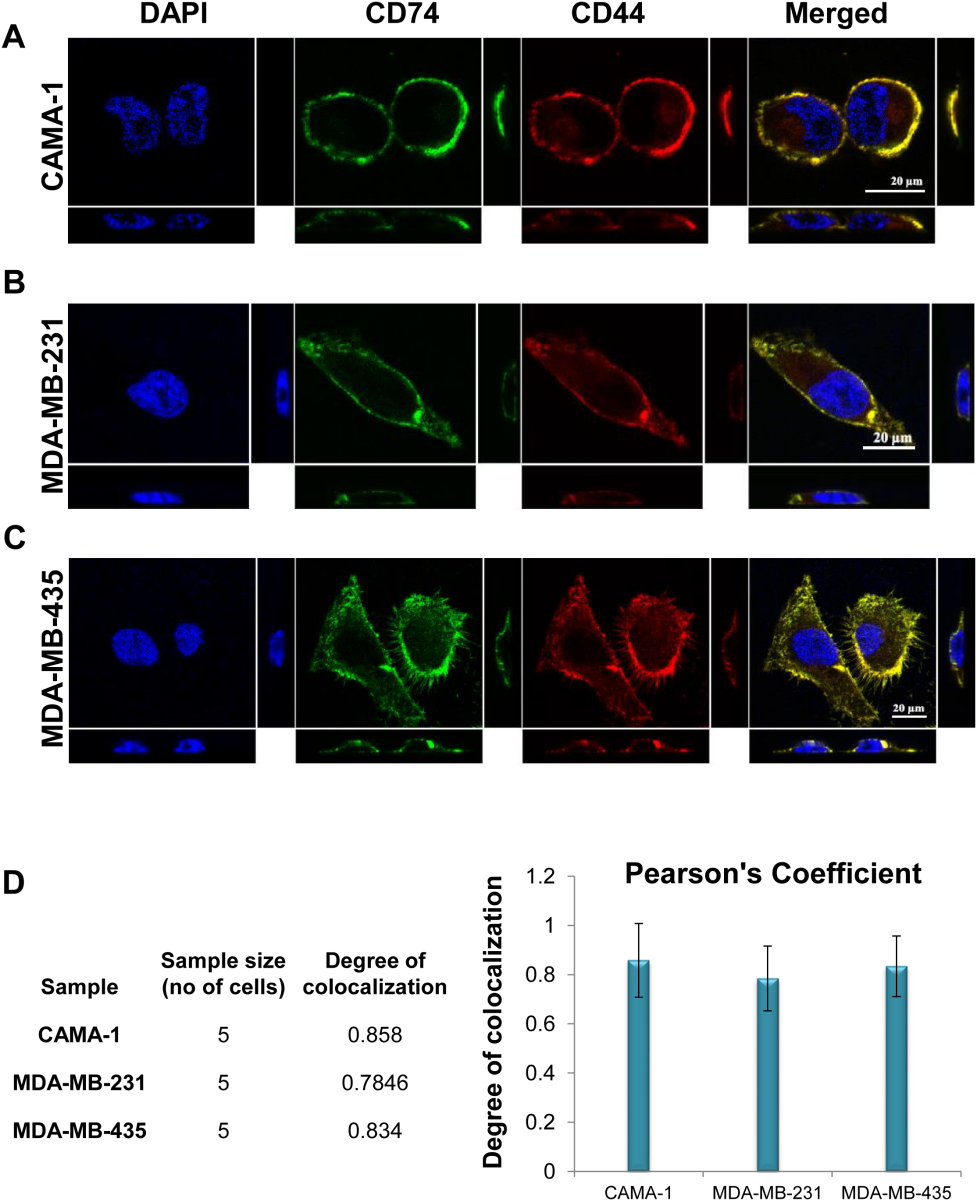
Colocalization of CD74 and CD44 intracellularly of **(A)** CAMA-1, **(B)** MDA-MB-231 and **(C)** MDA-MB-435 cells, determined by confocal microscopy analysis. Yellow/orange fluorescence reveals the potential colocalization of two antigens. 3D images were acquired in stack, with z-direction step size 0.14 μm using NIS element. Single-plane of z-stack is shown in three directions as xy, yz and zx. **(D)** Graphical representation of colocalization analysis based on Pearson’s correlation coefficient PCC of each cell. Data represent three different experiments. Figures depict representative samples from triplicate experiments. All data were statistically significant (*P*-values < 0.05).

To investigate whether CD74 and CD44 co-localize, both molecules were visualized separately at different wavelengths, appearing as green and red spots, respectively, in the image (Figure 5A-5C). The immunofluorescence-stained cells were imaged at single cell level. Data were analysed using the Pearson correlation coefficient (PCC). Figure 5D illustrates the colocalization of CD74 and CD44 in each cell line compared to the negative control, comprising colocalization of DAPI against FITC. For more accuracy, 3D images were acquired in stack in the z-direction and segmented by NIS elements to calculate the exact degree of colocalization of CD74 and CD44 molecules against the total volume in each of the images (Figure 5A, and 5C). The three breast cancer cell lines exhibited yellow/orange fluorescence in single cell images, signifying closely associated CD74 and CD44 receptors (Figure 5A-5C, panel at the far right). PCC values ranged from 0.784 to 0.858, reflecting distributions of probes that are correlated with one another, indicative of colocalization.

### Interaction of CD74 and CD44

**Figure 6:**
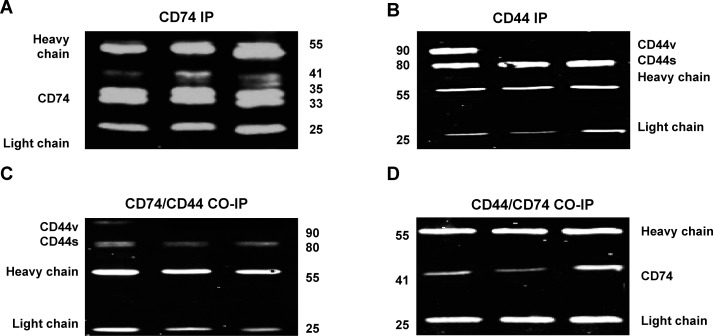
Co-immunoprecipitation (Co-IP) to study the interaction of CD74 and CD44 in CAMA-1, MDA-MB-231 and MDA-MB-435 cell lines **(A, B)** IP was subjected to pull down either CD74 or CD44. The blots were probed with either mouse anti-CD74 or anti-CD44 antibodies. The antibody (Ab) heavy and light chain bands are indicated so that Ab heavy and light chain fragments can be observed at approximately 55 and 25 kDa, respectively. **(C, D)** Co-IP was applied to study the interaction of CD74/CD44 and CD44/CD74. The Co-IP of CD74/CD44 confirmed that all CD44 isoforms, including CD44s and CD44, interact with CD74. However, Co-IP of CD44/CD74 confirmed that only p41 of CD74 interacts with CD44. Figures depict representative samples from duplicate experiments displayed as composites of multiple co-immunoprecipitation.

To further demonstrate interaction between CD74 and CD44, Co-IP was carried out. An interaction occurred between CD74 and CD44 in cell lysates from CAMA-1, MDA-MB-231 and MDA-MB-435 cells (Figure 6B). It was also revealed that, in CAMA-1 cells, CD74 interacts with both CD44s and CD44v isoforms. MDA-MB-231 and MDA-435 cells expressed only CD44s, which interacted with CD74. To test whether CD44s and CD44v interact with all isoforms of CD74 we performed Co-IP experiments with lysates of CAMA-1, MDA-MB-231 and MDA-435 cells, using either anti-CD74 or anti-CD44. The results obtained show that CD44s and CD44v bind p41 CD74 alone (Figure 6C).

## DISCUSSION

**Figure 7:**
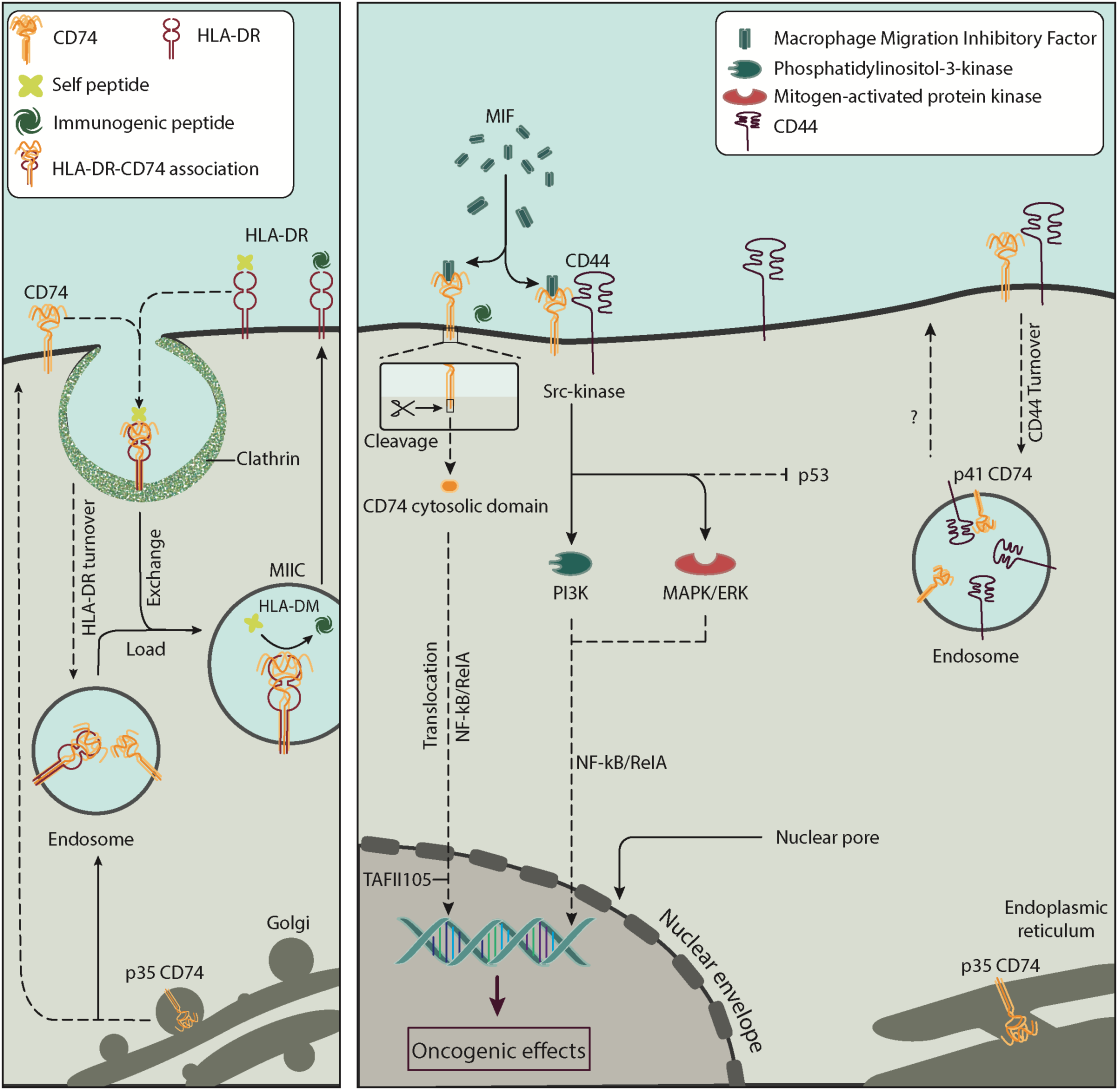
The interaction between CD74 and CD44 on the surface of cells CD74 is a single-chain type II integral membrane protein monomer; its expression is linked to the biosynthesis of HLA-DR, as shown in the left panel. CD74 associates with HLA-DR and is recycled into clathrin vesicles for peptide editing by HLA-DM. Cell-surface CD74 functions as a ligand for the cytokine MIF, as shown on the right panel. CD74 loaded with MIF interacts with CD44, a cell surface type I glycoprotein expressed on most cell types including cancer cells. Upon interaction CD44 activates the proto-oncogene tyrosine-protein kinase Src signal transduction pathway leading to NF-κB post-transcriptional regulation of genes. The role of CD74 and CD44 interaction in the turnover of these molecules is currently unknown. Acronyms: CD74, cluster of differentiation 74; CD74-ICD, cluster of differentiation 74 cytosolic domain; CD44, cluster of differentiation 44; HLA-DR, human leukocyte antigen-DR; HLA-DM, human leukocyte antigen-DM; MIIC, MHC class II compartment; MIF, macrophage migration inhibitory factor; MAPK/ERK, mitogen-activated protein kinase/extracellular signal-regulated kinase signalling cascade; NF-κB, nuclear factor κB; NF-κB/RelA, RelA subunit of nuclear factor κB; PI3K, phosphatidylinositol 3-kinase; TAFII105, TBP-associated factor.

We report the physical association of CD74 and CD44 and consider their likely function in breast cancer cells. Figure 7 summarize known interactions associated with CD74 and CD44 and the present results. Current data suggest that co-expression of CD74 and CD44 can play a significant role in the pathogenesis of various solid tumours [22, 33, 34]. No direct quantification has yet been conducted on the association of the two molecules. MIF-induced ERK1 and ERK2 kinase phosphorylation requires CD74 and co-expression of full-length CD44 [21]. MIF also interacts with the p53 tumor suppressor, inhibiting p53-responsive gene activation and apoptosis [35, 36]. To facilitate signaling, MIF binds to the extracellular domain of macrophage and B cell CD74 [17, 34]. However, CD44 is required for MIF signal transduction, due to lack of direct signalling via the cell-surface domain of CD74 [37]. When CD74 forms a complex with CD44, CD74 is modified by the addition of chondroitin sulfate, which is essential for the MIF-induced signaling cascade [37, 38]. It has been suggested that the CD74-MIF-CD44 complex initiates a pro-survival signal leading to reduced apoptosis and increased cell proliferation [21, 39].

Because CD74 and CD44 are both involved in MIF-mediated signalling, CD74 is considered a potential therapeutic target in cancer [24]. Therefore it is relevant to quantify the association of CD74 and CD44 in cancer cells. Meyer-Siegler et al. Showed that CD44 binds to p35 CD74 in bladder cancer cells, the isoform suggested to be involved in antigen presentation [33, 40]. Meyer-Siegler et al. Found that prostate cancer loses expression of CD44s and over-expresses almost entirely variant forms, which arise from alternate splicing of exons 7-10 [22]. This indicated potential involvement of CD44v in MIF-CD74-mediated signal transduction in prostate cancer. However, human benign prostate hyperplasia epithelial cells (BPH-1) and LNCaP prostate cancer cells do not express CD74 on the cell surface. For this reason, both cell lines do not interact with CD44. LNCaP cells, however, have been demonstrated to interact with MIF [22].

We show that CD74 and CD44 molecules are detectable by flow cytometry in CAMA-1, MDA-MB-231 and MDA-MB-435 breast cancer cells. Intracellular expression of CD74 and CD44 was detected by confocal microscopy. It was found that the level of expression of surface CD44 was significantly higher than that of CD74. High levels of CD74 expression might render tumors less immunogenic by blocking the MHC class II peptide-binding cleft, preventing binding of antigenic peptides for presentation to CD4+ lymphocytes [1, 41]. Indeed, several studies have shown that the level of CD74 expression is proportionality associated with tumor grade [41]. Surface CD74 is a prerequisite for the internalization of HLA-DR:Ii complexes, which are targeted to early endosomes [9]. However, changes in CD74 surface expression do not necessarily correlate with the level of this internalization activity. It has been shown that CD44 expression is associated with a high rate of cell division and proliferation status [28].

Western blotting revealed that CAMA-1, MDA-MB-231 and MDA-MB-435 cells express two isoforms of CD74, p35 and p41. CAMA-1 cells were observed to express CD44s and one CD44 variant isoform, at molecular weights of 80 and 90 kDa, respectively. In contrast to our data, Verjans et al. found that MDA-MB-231 and MDA-MB-468 cells express only one isoform of CD74 [34]. Similarly, Metodieva et al. Found that MDA-MB-435 express one CD74 isoform [42]. Two small CD74 isoforms, p33 and p35, are likely to be involved in regulating class II MHC antigen presentation while the p41 isoform may play an important role in T cell selection in the thymus [43, 44].

In terms of its function, the human-specific p35 CD74 isoform is the most enigmatic CD74 isoform. Use of an alternative translation initiation site in p35 adds R-X-R, an ER retention motif. It is thought that the R-X-R motif is blocked when MHC II binds CD74, permitting egress of MHC II/CD74 complexes from the ER. Mouse transgenic studies by Genève et al. Utilizing mice that lack endogenous CD74, indicate that p35 retains the antigen presenting functions of other CD74 isoforms in T-cell-positive selection and peripheral maintenance [40]. This suggested that p33 and p35, together, facilitate antigen presentation, and that this process does not require the co-expression of any other CD74 isoform [1, 40]. If so, the p35 isoform could substitute for other CD74 isoforms if they were absent.

Western blot analysis showed that CAMA-1 cells co-express CD44s and CD44v. Jung et al. Showed that some breast cancer cell lines, such as MDA-MB-468 and SUM149, express several isoforms of CD44 [45]. It is possible that CD44 expression in breast cancer is associated with highly aggressive breast tumour subtypes or highly invasive breast cancer cells [46].

We have studied immortalized normal breast luminal cells (226LDM). Confocal microscopic images showed that these cells did not express CD74, but did express intracellular and cell surface CD44. The absence of CD74 in 226LDM cells was anticipated, since CD74 has, so far, only been detected in antigen-presenting cells, including B cells, monocytes, macrophages, dendritic cells and Langerhans cells, and in inflammatory cells, including those detected in cancers [47]. The presence of CD44 in normal breast cells is expected, since CD44 has a wide range of functions in normal tissues. Our results showed very low CD44 surface expression in 226LDM cells with higher intracellular expression.

Confocal microscopy analysis revealed that CD44 and CD74 are highly colocalized on endosomal membranes in CAMA-1, MDA-MB-231 and MDA-MB-435 cells. The colocalization results obtained by correlation coefficient and total segmented volume indicated that CAMA-1 cells possess the greatest number of colocalized CD74 and CD44 molecules, followed by MDA-MB-435 and MDA-MB-231 cells. However, the degree of colocalisation in all three tumour cell groups was comparable.

colocalization between CD74 and CD44 may suggest that both are involved in signalling pathways along with MIF. CD44 has been found to form an active signalling complex with ErbB2 [48]. It also can anchor matrix metalloprotease-7 to the membrane, resulting in activation of ErbB4 [49].

One mechanism by which CD44 reaches intracellular sites is processing of its transmembrane form by consecutive cleavages by metalloproteases and c-secretase to generate a soluble extracellular domain and an intracellular domain. The latter is translocated to the nucleus where it activates transcription of genes involved in cell migration and attachment [50, 51]. Pályi-Krekk et al. Observed that hyaluronan oligosaccharide, epidermal growth factor and heregulin induce CD44 shedding, which was accompanied by endocytosis of full length CD44 and intramembrane cleavage, with accumulation of its intracellular domain in the cytosol [52].

In B cells, CD74 is internalized into endocytic compartments. Within them, intramembrane cleavage releases its cytosolic intracellular domain, CD74-ICD, which then enters the nucleus, activates NF-κB p65/RelA, and controls the differentiation of B cells through the TAF_II_105 coactivator [4, 53]. Studying HT-29 colon adenocarcinoma cells, Moldenhauer et al. Showed that CD74/Ii utilizes two internalization motifs to internalize HLA-DR molecules, to deliver HLA-DR:Ii to early endosomes [9].

Upregulation and colocalization of CD74 and CD44 was observed in a preliminary pilot study using Grade III triple negative cancer cells, matched for size and nodal status (Al Ssadh Hussain, unpublished data). The present study has clearly demonstrated the occurrence of quantifiable colocalization between CD74 and CD44 in the plasma membrane of the breast cancer-derived cell lines CAMA-1, MDA-MB-231 and MDA-MB-435, suggesting interaction of these two molecules in breast cancer cells. We also found that CD44 in MDA-MB-435, CAMA-1 and MDA-MB-231 cells displayed higher expression than CD74. The level of expression of cell surface CD74 was higher in MDA-MB-435 cells compared to CAMA-1 and MDA-MB-231 cells. Co-IP results confirmed that CD44 interacts only with p41, the most abundant isoform of CD74.

The procedure used herein for identifying receptor pair associations can be applied to studying protein-protein relationships in other cancers and could be used to assemble antibodies to monitor cancer progression. Although about 10-20% of breast cancers are triple negative, it would be of interest to study CD74 and CD44 in a cohort of breast cancer cells tested negative for estrogen receptors, progesterone receptor, and HER2. In summary, measuring the co-expression levels of CD74 and CD44 could potentially be used as a ‘biomarker signature’ for examining different stages of breast cancer.

## MATERIALS AND METHODS

### Cell lines and cell culture

Three human mammary gland cell lines were used, CAMA- 1, MDA-MB-231 and MDA-MB-435, all derived from malignant pleural effusion. The CAMA-1 and MDA-MB-435 cell lines were maintained in RPMI 1640 medium (LONZA-Belgium), supplemented with 10% (v/v) fetal calf serum (FCS; Imperial Laboratories, Andover, UK). In line with numerous studies, we used the MDA-MB-435 cell line as a model for breast cancer, but acknowledge recent concerns that it might be derived from the M14 cell line MDA-MB-435. The MDA-MB-231 cell line was maintained in D-MEM (high glucose), supplemented with 10% FCS. 226LDM immortalized normal breast luminal cells (kindly provided by Elena Klenova, School of Biological Sciences, University of Essex), were used as positive control cells. Raji cells (human negroid Burkitt's lymphoma) and HeLa cells (human cervical cancer), expressing high levels of CD74 and CD44, respectively, served as additional positive controls. The 226LDM cell line was maintained in Dulbecco's Modified Eagle's Medium (DMEM)/F-12, supplemented with 10% FCS, 5 mg/ml gentamicine, 5 μg/m insulin, l ng/ml hydrocortine, 20 ng/ml epidermal growth factor and 20 ng/ml cholera toxin. Raji and HeLa cells were cultured in RPMI 1640 (LONZA-Belgium) containing 10% FCS and cultured in a humidified atmosphere of 5% CO_2_ at 37°C. All media used for this study were purchased from PAA Laboratories GmbH (Pasching, Austria).

### Reagents

The monoclonal primary antibodies mouse anti-human CD74 (clone: By2) and mouse anti-human α-tubulin (clone: TU-02) were purchased from Santa Cruz Biotechnology, USA. Mouse antibody CD44 (clone: 156-3c11) was purchased from Cell Signaling Technology, USA. Rabbit anti-human β-actin (clone: poly 6221) was purchased from BioLegend, UK. The secondary antibody used for flow cytometry was a goat anti-mouse antibody conjugated with the fluorophore FITC (clone: poly4053) and was purchased from Bio-legend, UK. The secondary antibody used for Western blotting was either goat anti-mouse (IRDye 800CW) or goat anti-rabbit (IRDye 680 LT), purchased from LI-COR Biosciences. Finally, Alexa 488 (green) and Alexa 555 (red) antibodies were purchased from Life Technologies, UK.

### Flow cytometry analysis

Cell lines were lifted with Accutase (Sigma-Aldrich) and 1 × 10^6^ cells were used per sample. Monoclonal antibodies By2 (anti-CD74) and 156-3c11 (anti-CD44) were employed in indirect immunofluorescence staining. Cells were preincubated with saturating concentrations of primary antibody, followed by washing and labeling with FITC-conjugated goat anti-mouse IgG (Bio-legend). For cell-surface staining, cells were fixed with 4% formaldehyde solution, and washed with 1X phosphate-buffered saline (PBS). The cells were then blocked with blocking buffer (PBS/0.1% BSA, bovine serum albumin) and washed in PBS. Primary and secondary antibodies were diluted with 0.1% BSA in PBS. Cells were sorted on a BD FACSAria and analyzed by FlowJo 8.8.6.

### Western blotting and immunodetection

Cells were lysed with CelLytic reagent (Sigma-Aldrich) and total protein concentration was determined by Bradford assay. The total cell lysate was separated on a 12% SDS-PAGE gel. A total of 40 μg protein was loaded per well, and, after electrophoresis, protein was transferred to a polyvinylidene fluoride (PVDF) membrane (Immobilon-FL, Merck Millipore, Merck KGaA, Darmstadt, Germany). Membranes were blocked with 5% skimmed milk in PBS-Tween-20 (Sigma) for 1 h at room temperature and incubated in anti-CD74 (clone: By2) at a concentration of 1:200, and anti-CD44 (clone: 156-3C11) at a concentration of 1:1000. As a control, an alpha subunit-specific tubulin mouse monoclonal antibody was used to probe the cell extracts at a concentration of 1:200, followed by washing in PBS-Tween-20 for 30 min. The membranes were then incubated with IRDye 800CW donkey anti-mouse IgG (LI-COR Biosciences, Lincoln, NE, USA) at a concentration of 1:1000 for 1 h followed by washing in PBS-T for 30 min. Signals were detected using the ODYSSEY Infrared Imaging System (LI-COR Biosciences). Fermentas PageRuler^™^ Plus Prestained Protein Ladder (Thermo Fisher Scientific, Waltham, MA, USA) was used in order to estimate the molecular weight of the respective protein bands. To evaluate the differences in protein loading during the experiment, the percentage of expression was calculated after the intensity of each band was adjusted according to its respective β-actin band intensity using Image Studio Lite software (LI-COR Biosciences).

### Immunofluorescence

In preparation for confocal immunofluorescence microscopy for studying colocalization between CD74 and CD44 intracellularly, CAMA-1, MDA-MB-231 and MDA-MB-435 cells were cultured in LabTek 8-well chambers (Thermo Fisher Scientific) at a density of 6 × 10^3^ cells per well for two days. Following this they were seeded. The cells were fixed with 4% paraformaldehyde for 20 min on ice. For immunofluorescence staining, all procedures were carried out at ambient temperature. Cells were permeabilized with 0.1% Triton X-100 and then blocked with 2% (w/v) BSA prepared in 1X PBS for 1 h at room temperature. For single staining of each antigen, cells were incubated with anti-CD74 (clone: By2) at a concentration of 1:500, and anti-CD44 (clone: 156-3C11) at a concentration of 1:400 for 1 h, followed by three washes with PBS. Secondary antibody, anti-mouse IgG conjugated with Alexa Fluor® 488 or Alexa Fluor® 555 (Invitrogen, Carlsbad, CA, USA), was used at a dilution of 0.25 μg/100ml for 1 h. For double staining, cells were blocked again with 2% BSA and the staining process was repeated for each desired pair. Cells were incubated with a primary antibody followed by a secondary antibody. CD74 was labeled with FITC Alexa Fluor 488 (green) and CD44 was labeled with Alexa Flour 555 (red). Colocalization, i.e. the overlap of CD74 with CD44, was assessed by merging green and red channels and the Pearson's correlation coefficient was used to analyse the degree of colocalization. The scale lay between -1 and 1, where 1 stands for colocalization, -1 stands for negative colocalization and 0 stands for no colocalization. 4′, 6-diamidino-2-phenylindole di-hydrochloride (DAPI) counter stain (Vector Laboratories, Burlingame, CA, USA) was used at a 1:250 dilution. Cells were thoroughly washed with PBS, the chambers removed, and the slide was mounted with anti-fade mounting medium (Vector Shield) covered with a cover slip (Chance proper LTD, West Midlands, England) and sealed with rubber cement (Fixogum Rubber Cement, Marabu, Germany).

### Quantitative colocalization analysis of confocal fluorescence microscopy images

To investigate whether CD74 and CD44 co localize, a high-precision single-cell bioimaging protocol was employed, previously developed by our research group [54]. The Pearson correlation coefficient of was used for quantitative analysis of colocalization [54, 55]. PCC provides a statistic able to measure the overall association of two probes in an image. It also indirectly measures quantity, i.e. the fraction of one protein that colocalizes with another protein. A Nikon A1Si confocal microscope (Nikon Instruments Inc.) with a plan-apochromatic VC1.4 N.A. 60x magnifying oil-immersion objective was used for image acquisition. Images were acquired in three channels, using one-way sequential line scans. DAPI was excited at 398.7 nm with laser power 1.6 arbitrary units, and its emission was collected at 450 nm with a PMT gain of 86. Alexa Fluor 488 was excited at 488 nm with laser power 5.8; its emission was collected at 525 nm with a PMT gain of 117. Alexa Fluor 555 was excited at 560.5 nm with laser power 3.7, and its emission was collected at 595 nm with a PMT gain of 98. The scan speed was ¼ frames/s (galvano scanner). The pinhole size was 35.76 μm, approximating 1.2 times the Airy disk size of the 1.4-NA objective at 525 nm. Scanner zoom was centered on the optical axis and set to a lateral magnification of 60 nm/pixel. Axial step size was 105 nm, with 80-100 image planes per z-stack.

### Image processing

NIS-Elements software (version 3.21.03, build 705; Nikon Instruments Inc.) was used for image processing. CD74 (green) and CD44 (red) channels were segmented using regional maxima detection tools followed by manual threshold. The generated binary areas were visually inspected; the overlap of the green and red channels, which were generated by the overlay tool, resulted in a new layer (yellow) that represents the intersection of CD74 and CD44. Finally, automated volume measurement was carried out for CD74 and CD44, and their intersection was measured by a volume measurement tool [54, 56].

### Immunoprecipitation and co-immunoprecipitation

Cells were lysed with the RIPA Lysis Buffer System (Santa Cruz Biotechnology, USA) and total protein concentration was determined by Bradford assay. 1 mg/ml of each sample was incubated overnight with 4 μg of anti-CD74 (clone: By2), at 4°C. Following that, 40 μl of protein A/G PLUS-agarose (Santa Cruz Biotechnology, USA) was added. To promote immunoglobulin binding, this solution was kept overnight on a rotator at 4°C. The samples were spun down for 30 s and the supernatant was discarded. Beads were then washed twice using PBS and the samples were boiled at 100°C after adding 50 μl of SDS-PAGE sample loading buffer containing di-thiothreitol. Then 20 μl of each sample was loaded in each well of the gel and was left for 1 h and 30 min at 120 V. After electrophoresis, protein was transferred to a PVDF membrane. Membranes were blocked with 5% skimmed milk in PBS-Tween-20 (Sigma) for 1 h at room temperature and incubated in either anti-CD74 (clone: By2) at a concentration of 1:200, or anti-CD44 (clone: 156-3C11) at a concentration of 1:1000, and followed by washing in PBS-T for 30 min. The membranes were then incubated with secondary antibody (IRDye 800CW donkey anti-mouse IgG; LI-COR Biosciences, Lincoln, NE, USA) at a concentration of 1:1000 for 1 h, followed by washing in PBS-T for 30 min. Signals were detected using the ODYSSEY Infrared Imaging System (LI-CORBiosciences).
